# Control of *Aedes aegypti* Breeding: A Novel Intervention for Prevention and Control of Dengue in an Endemic Zone of Delhi, India

**DOI:** 10.1371/journal.pone.0166768

**Published:** 2016-12-05

**Authors:** B. N. Nagpal, Sanjeev Kumar Gupta, Arshad Shamim, Kumar Vikram, Aruna Srivastava, N. R. Tuli, Rekha Saxena, Himmat Singh, V. P. Singh, V. N. Bhagat, N. K. Yadav, Neena Valecha

**Affiliations:** 1 National Institute of Malaria Research (NIMR), New Delhi, India; 2 Municipal Corporation of Delhi (MCD), Delhi, India; 3 Department of Health Research (DHR), Ministry of Health & Family Welfare, Delhi, India; Institut Pasteur, FRANCE

## Abstract

**Background and objective:**

The study is based on hypothesis that whether continuous entomological surveillance of *Ae*. *aegypti* and simultaneous appropriate interventions in key containers during non-transmission (December–May) months would have any impact on breeding of *Aedes* and dengue cases during the following transmission months (June–November). The impact of the surveillance and intervention measures undertaken during non-transmission months were assessed by entomological indicators namely container index (CI), house index (HI), pupal index (PI) and breteau index (BI).

**Methods:**

A total of 28 localities of West Zone of Delhi with persistent dengue endemicity were selected for the study. Out of these localities, 20 were included in study group while other 8 localities were in control group. IEC and various *Aedes* breeding control activities were carried out in study group in both non-transmission and transmission season whereas control group did not have any such interventions during non-transmission months as per guidelines of MCD. These activities were undertaken by a team of investigators from NIMR and SDMC, Delhi. In control group, investigators from NIMR carried out surveillance activity to monitor the breeding of *Aedes* mosquito in localities.

**Results:**

Comparison of baseline data revealed that all indices in control and study group of localities were comparable and statistically non-significant (p>0.05). In both study and control groups, indices were calculated after pooling data on seasonal basis, i.e., transmission and non-transmission months for both years. The test of significance conducted on all the four indices, i.e., HI, PI, CI, and BI, revealed a significant difference (p<0.05) between the study group and control group during transmission and non-transmission months except in HI. Due to consistent intervention measures undertaken in non-transmission months in study group, reduction in CI, HI, BI and PI was observed 63%, 62%, 64% and 99% respectively during transmission months as compared to control group where increase of 59%, 102%, 73% and 71% respectively. As a result of reduction in larval indices, no dengue case (except one NS1) was observed in study group, whereas 38 dengue cases were observed in control group.

**Conclusion:**

Through this pilot study, it is concluded that proper intervention in non-transmission season reduces vector density and subsequently dengue cases in transmission season.

## Introduction

Dengue, a fast spreading vector borne disease is endemic in more than 100 countries with half of world’s population living in area at risk of this disease [1]. Heaviest burden of dengue is reported by Asia Pacific countries including India with 1800 million people at risk of dengue infection [2]. World Health Organization-South East Asia (WHO-SEA) has placed India in ‘Category A’ in terms of dengue endemicity as being a major public health problem, leading cause of hospitalization death among children, hyper-endemicity with all the four serotypes in circulation and is spreading to rural areas along with other SEA countries viz. Bangladesh, Indonesia, Maldives, Myanmar, Sri Lanka, Thailand and Timor-Leste [3].

Population growth, unplanned urbanization and poor water management systems leading to frequent water shortages and storage practices have promoted breeding sites for the *Aedes* mosquito [4] which primarily breeds in domestic water storage containers in and around human dwellings [5]. In the absence of dengue vaccine and drugs to cure the disease till date, vector control is the only option available to prevent outbreak of dengue [6]. Urban areas with high-density of water storage receptacles are suitable for breeding of *Aedes* mosquitoes [4]. In most of these areas small number of *Aedes* breeding habitats exist even during the adverse months of the year and consistently serve as the primary producers of *Ae*. *aegypti*, referred as “Key Containers” [7] which are region specific for *Aedes* breeding [8]. Key containers in Philippines include plastic & metal drums and plastic containers [9] while it is roof gutters in Australia [10]. In India, cement tanks and plastic containers were identified as major breeding habitats of *Aedes aegypti* [11–12]. In the capital city Delhi, India overhead tanks and curing tanks were identified as key containers of *Aedes* breeding [13].

It is well known fact that dengue infection commonly occurs during or after rainfall (wet season) as an outcome of increase in vector population [14]. As the rainfall patterns vary from place to place, the entire year can be classified in two parts i.e. transmission (wet) and non-transmission (dry) season. Based on rainfall *Aedes* breeding and dengue cases are observed in Delhi during June to November period, referred as transmission season whereas December to May as non-transmission season for dengue [13].

As per census 2011, Delhi has a population of about 16.75 millions with about 4.6 million households. Delhi Municipal Corporations and New Delhi Municipal Corporation are responsible for implementing vector control strategies in Delhi & New Delhi areas respectively.

In view of the high risk of dengue transmission in Delhi, proactive surveillance and intervention measures are required to control the potential outbreaks of dengue. An attempt has been made in the study to identify the key containers (primary containers) for *Aedes* breeding during non-transmission months and assess the impact of interventions undertaken on dengue transmission in wet months.

## Material & Methods

### Study Area

**Fig 1:**
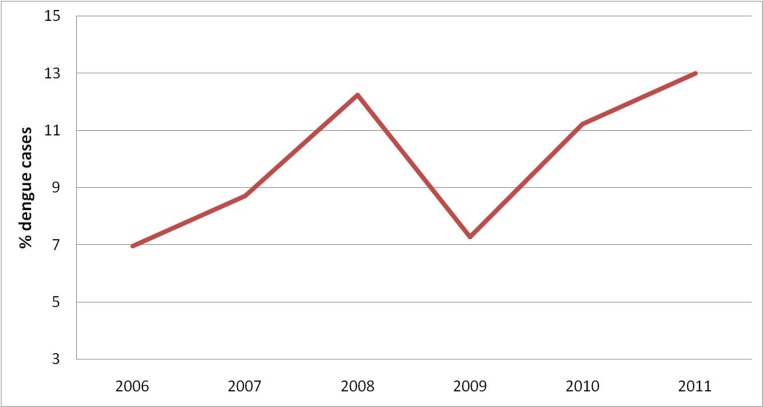
Percent cases in West Zone of Delhi (2006–2011).

The Municipal Corporation of Delhi (MCD), India is among the largest municipal bodies in the world providing civic services to more than the estimated population of 11 million citizens of Delhi. The entire MCD area is divided into 12 zones spread across three smaller municipal corporations viz. North, South and East Delhi. West zone of Delhi falls under South Delhi Municipal Corporation (SDMC), consists of 36 wards and 277 localities with a population of about 2.5 million. The density of the SDMC is 19,625 inhabitants per square kilometre. As per MCD records, 7% - 13% of the total dengue cases reported by SDMC, Delhi during the period 2006 to 2011 (Fig 1) were contributed by west zone. In view of the persistent reporting of dengue cases by west zone of SDMC Delhi, it was selected as the study site. The study was carried out during the period July 2012 to May 2014 in collaboration with SDMC, Delhi.

The primary objective of the study was to test a hypothesis which was formulated from an earlier study undertaken in Delhi Municipal Corporation [15], whether continuous entomological surveillance of *Ae*. *aegypti* and simultaneous appropriate interventions in key containers during non-transmission (December–May) months would have any impact on breeding of *Aedes* and dengue cases during the transmission months that follow (June–November). The impact of the surveillance and intervention measures undertaken during non-transmission months was assessed by entomological indicators namely container index (CI), house index (HI), Pupal Index (PI) and breteau index (BI) in the following transmission months.

In Delhi, *Aedes* breeding sites have been classified in 9 major categories: 1) Overhead tanks (plastic and cemented tanks fixed on the terrace of houses); 2) water storage containers (containers used for water storage purposes); 3) curing tanks (cemented tanks built at construction sites); 4) coolers; 5) flower pots; 6) bird pots; 7) mud pots 8) pits (cemented) and 9) solid wastes (dump tyres, disposable plastic/thermo cool/paper glasses etc.). As per the results of earlier study carried out in Delhi during 2007 to 2012 (Dengue Bulletin, 2015), Over Head Tanks (OHTs) and Curing Tanks, which remained persistently positive throughout the year and worked as mother foci, were identified as primary containers for *Ae*. *aegypti* breeding in Delhi.

The impact of surveillance and intervention measures that were undertaken in non-transmission months was assessed by change in key entomological indicators namely house index, container index, pupal index and breteau index during the transmission months of the study period.

### Study Design

The study is quasi experiment parallel group design. A total of 28 localities with persistent dengue endemicity were selected out of the total 277 localities of west zone, Delhi. Out of these localities, 20 were included in study group while other 8 localities were included in control group by convenient sampling in such a way that localities under control group were at least 2 km distant from the study group. The following interventions were carried out in study group during non-transmission season; 1) fortnightly surveillance of key containers; 2) accelerated community mobilization by organizing meetings of representatives of resident welfare associations (RWAs); trade unions; school teachers; school children; municipal councilors and local opinion leaders including live demonstration of different stages of *Aedes* mosquito in study localities; 3) closing of OHTs by proper lids or other material available locally; 4) regular use of temephos granules with a dose of 1 ppm in OHTs and curing tanks and 5) source reduction. These activities were undertaken by a team of investigators from NIMR and SDMC, Delhi. Control group did not have any such interventions during non-transmission months as per guidelines of MCD. However, investigators from NIMR carried out surveillance activity on 3 monthly basis to monitor the breeding of *Aedes* mosquito in localities under control group.

All the activities undertaken in study group were continued during transmission months, however, in control group conventional intervention and entomological surveillance was carried by domestic breeding checkers engaged by SDMC.

For each locality, container-wise data was pooled on monthly basis to calculate indices like container index, house index and pupal index using the following formulae;

**Container Index (CI)** = No. of *Aedes* larvae Positive containers x 100 / No. of containers with water inspected
**House Index (HI)** = No. of houses positive for *Aedes* larvae x 100/ No. of houses inspected
**Pupal Index (PI)** = No. of Pupae x 100 / No. of houses inspected
**Breteau Index (BI)** = Number of positive containers x 100 / No. of Houses inspected

Although we have considered all the four indices to gauge the magnitude of the transmission, however, these have advantages and limitations in interpretation of their public health importance. PI has been considered as the most sensitive parameter for assessing the effectiveness of the intervention

### Statistical Analysis

The data were entered in Excel 2007 and statistical analysis was done using SPSS software package (version 20). Among the test conducted between indices the control value was taken as expected value.

### Proximity Analysis

Location of surveyed houses and houses with dengue cases were mapped using GARMIN’s eTrex 30 GPS whereas ArcGIS 9.3 software was used to create buffer zone around surveyed houses

### Ethics Statement

This study was carried out in strict accordance with the recommendations of Research Advisory Committee and Scientific Advisory Committee of NIMR. The study was carried out in collaboration with South Delhi Municipal Corporation (SDMC), competent authority in Delhi, India and therefore, no specific permissions were required to conduct breeding survey. Authors hereby confirm that this field study did not involve endangered or protected species.

## Results

Surveillance and intervention of *Aedes* breeding was carried out in 7479 houses (≈37000 population) in study group on fortnightly basis, while in control localities surveillance of *Aedes* was conducted in 1238 houses (≈6200 population) on once in three months interval. Baseline survey was performed in the month of July’ 2012 and following larval indices were recorded in both groups of localities (Table 1).

**Table 1:** Baseline larval indices during the month of Jul, 2012.

Larval Indices	Study Group	Control Group	Chi^2^(p-value)
**House Index**	7.0(n = 2773)	6.8(n = 1242)	0.01 (p = 0.9203)
**Container Index**	3.6(n = 6142)	4.6(n = 1138)	0.22 (p = 0.6390)
**Breteau Index**	8.04	4.19	3.54 (p = 0.0599)
**Pupal Index**	11.54	11.91	0.01 (p = 0.9203)

As observed from the above table, all indices in control and study groups of localities were comparable and statistically non-significant (p>0.05) indicates that there was no difference between population of study and control group.

For the sensitization of the community in localities under study group, three workshops were organized with the objective of imparting information about dengue fever, its transmission and characteristics of the vector, the *Aedes* mosquito, and measures to control. The workshop was attended by the representatives of resident welfare associations, traders unions, school teachers, students and local municipal councilors. Besides interaction with the group, pamphlets which contained information on symptoms of dengue, breeding sites of *Aedes* mosquito and precautions needed to avoid *Aedes breeding* were also distributed among community.

During the study period, out of the 6877 OHTs surveyed each month, 185 OHTs with broken lids were covered properly with locally available material. Temephos with a dose of 1 ppm was introduced in 1213 containers, mainly OHTs, Coolers and Water storage containers at fortnightly basis. A total of 1408 containers, mostly mud pots and solid waste were emptied and scrubbed to remove/destroy the eggs of *Aedes* attached to the wet surface of the containers and community was advised to expose the container in sunlight for at least 2 hrs. However, no such intervention activity was undertaken in localities under control group.

### Impact of intervention on larval indices

In both study and control groups, indices were calculated after pooling the data on seasonal basis i.e. transmission and non-transmission months for both the years. The test of significance was applied on all the four indices i.e. HI, PI, CI, and BI between the study group and control group (Table 2)

**Table 2:** Comparison of study and control groups for larval Indices.

Period/Indices	Transmission Season			Non-Transmission Season		
	Study Group	Control Group	Chi^2^(p-value)	Study Group	Control Group	Chi^2^(p-value)
**2012–2013**						
House Index	3.49	4.50	0.23(p = 0.6315)	0.91	1.90	0.52(p = 0.4708)
Container Index	2.02*	12.41	8.70(p = 0.0032)	0.61*	5.97	4.81(p = 0.0283)
Pupal Index	4.91*	15.83	7.53(p = 0.0061)	0.22	1.85	1.44(p = 0.2301)
Breteau Index	4.48*	18.65	10.77(p = 0.0010)	1.11*	9.39	7.30(p = 0.0069)
**2013–2014**						
House Index	1.31*	9.10	6.67(p = 0.0098)	0.05*	5.70	5.60(p = 0.0180)
Container Index	0.75*	19.67	18.20(p = 0.0001)	0.03*	6.58	6.52(p = 0.0107)
Pupal Index	0.04*	27.10	27.02(p = 0.0001)	0.00	2.26	2.26(p = 0.1328)
Breteau Index	1.62*	32.27	29.11(p = 0.0001)	0.06*	11.11	10.99(p = 0.0009)

* p<0.05 statistically significant

A significant difference (p<0.05) was observed in larval indices between the study and control group localities during transmission and non-transmission months except in HI.

### Impact of intervention on individual breeding habitats

On comparing container index of different breeding habitats of 2012–13 with 2013–14 in study group, it was observed that in study group maximum reduction was observed in mud pots (77%) followed by overhead tanks (76%) and water storage plastic containers (67%). Curing tanks (6%) and solid waste (48%) were exception in study group where an increase in container index was observed (Table 3).

**Table 3:** Year-wise seasonal comparison of container index (CI) in different breeding habitats.

Season/Breeding Habitat	Study Group			Control Group		
	2012–13	2013–2014	% Change	2012–2013	2013–2014	% Change
**Non Transmission Season**						
OHT	0.34	0.02	93↓	8.33	8.62	3 ↑
Cooler	2.93	0.24	92↓	2.87	4.04	41 ↑
Curing tanks	12.94	10.26	21↓	19.05	21.43	13 ↑
Mud Pots	1.19	0.00	100↓	1.91	3.58	87 ↑
Solid Waste	2.50	0.00	100↓	6.67	13.33	100 ↑
Water Storage Containers	0.57	0.02	97↓	5.08	5.65	11 ↑
**Transmission Season**						
OHT	0.53	0.18	66↓	10.33	20.62	100 ↑
Cooler	4.87	2.53	48↓	10.45	15.61	49 ↑
Curing tanks	15.05	15.89	6 ↑	6.00	10.00	67 ↑
Mud Pots	3.76	1.03	73↓	33.33	20.97	37↓
Solid Waste	9.49	14.00	48 ↑	19.15	37.84	98 ↑
Water Storage Containers	1.91	0.74	61↓	13.21	19.90	51 ↑
**Total**						
OHT	0.42	0.10	76↓	9.09	12.92	42 ↑
Cooler	4.51	2.11	53↓	5.64	10.80	92 ↑
Curing tanks	14.11	15.15	7 ↑	12.63	30.13	139 ↑
Mud Pots	2.49	0.57	77↓	11.78	9.78	17↓
Solid Waste	7.47	11.29	51↑	16.13	30.77	91 ↑
Water Storage Containers	1.15	0.38	67↓	12.17	15.22	25 ↑

↓ Decrease ↑Increase

In contrast, breeding habitats showed increase in CI with maximum increase was observed in curing tanks (139%) followed by coolers (92%) and solid waste (91%). Mud pots (17%) were exception, in which decrease in CI was observed on comparing CI of 2012–13 with 2013–14 in control group (Table 3).

### Impact on dengue cases

A total of 2023 and 5572 dengue cases were recorded by MCD in Delhi during 2012 and 2013 respectively. West zone of Delhi was not exception and contributed 128 (6%) and 458(8%) of the total cases reported in year 2012 and 2013 respectively. However only one case (NS1 positive) was recorded from the study area during 2012 and 2013, whereas in control area a total of 38 dengue cases were reported in the year 2012 & 2013 (Table 4).

**Table 4:** Incidence of dengue cases recorded from Delhi, study group and control group.

	Delhi		Study Group			Control Group		
	2012	2013	2012	2013	Total	2012	2013	Total
Dengue Cases	2023	5572	0	1*	1*	35	3	38
Incidence ofcases / 1000^#^	0.12	0.32	0	0.03	0.03	5.65	0.48	6.13

* NS1

# Incidence of cases/1000 = No. of dengue cases reported x 1000 / Population

**Fig 2:**
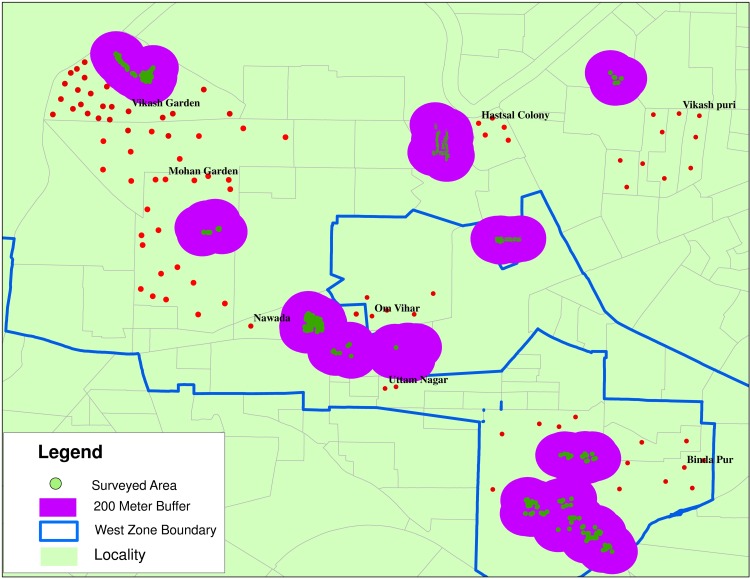
GPS location of dengue cases recorded and buffer zone around surveyed houses

A 200m buffer zone was created around surveyed houses and was compared using GPS coordinated of the residence of person affected with dengue case recorded from West Zone revealed that all dengue cases of the West Zone fall outside 200 m buffer zone i.e. the flight range of *Aedes* mosquitoes [16] (Fig 2). A buffer in Geographic Information System (GIS) is a proximity analysis tool used to create a zone around a map feature measured in units of distance. Buffering point data involves the creation of a circular polygon about the point of interest with radius of this circular polygon is called the buffer distance [17].

## Discussion

A study carried out in Brazil showed that targeting of the key containers resulted in decrease of the adult mosquito over time [18].However in the present study emphasis was given to target key containers during non-transmission season and the impact was assessed by recording of larval indices and dengue cases during transmission months.

Some of the studies did not find a significant correlation between larval indices viz. HI, BI, CI and PI and dengue transmission/ dengue outbreak prediction[19], [20],whereas other reported that either of these indices or their combination has importance in prediction of the risk of dengue transmission/dengue outbreak[21–23]. It has been suggested that Container Pupal Index and proportion of containers positive with pupae be taken the basis for vector surveillance and disease control [24].In the present study, due to consistent intervention measures undertaken in non-transmission months in study group reduction in CI, HI, BI and PI was observed -63%, 62%, 64% and 99% respectively during transmission months as compared to control group where increase in CI, HI, BI and PI was recorded—59%, 102%, 73% and 71% respectively. As a result of reduction in larval indices, one dengue case (NS1 positive) was reported in study group, whereas 38 dengue cases were reported in control group which clearly shows a positive correlation between larval indices and dengue transmission.

In study group *Aedes* breeding habitats OHTs, coolers, mud pots and water storage containers showed reduction in container index by76%, 53%, 77% and 67% respectively. On the contrary, curing tanks and solid waste were found exception where increase in container index was noted by 7% and 51% respectively. While in control group a substantial increase in container index was reported in OHTs (42%), coolers (92%), curing tanks(139%) and solid waste(91%). Out of six categories of containers, curing tanks and solid waste are the only two peri-domestic water containers that recorded increase in container index in study group of localities. This may be attributed to the fact that construction is round the year activity in Delhi and even after the completion of construction work, the tanks are usually not demolished, and therefore, become a constant source of *Aedes* breeding. Solid waste is defined as garbage or discarded material which is no longer useful or required after the completion of a process. The per capita generation rate of municipal solid waste in India ranges from 0.2 to 0.5 kg/ day andDelhi witnessed itself at the upper limit i.e. 0.475 kg/day[25]. Due to lack of proper disposal system of solid waste in Delhi, it is indiscriminately dumped everywhere [26], [27], remaining a major threat for the increase in the population density of the *Aedes* mosquitoes in urban and industrial towns [28], [29]. Responsibility of solid waste management remains primarily with the municipal bodies [30],therefore, reduction in solid waste and its proper disposal is utmost important for reducing *Aedes* breeding and density in Delhi.

The major achievement of the study was to demonstrate that by community participation and social mobilization, coupled with regular active surveillance of key habitats for *Aedes* breeding during dry and wet months of the year and simultaneous interventions like putting proper lids on OHTs, regular emptying of water containers and their cleaning, treatment of coolers and curing tanks with temephos and proper and safe disposal of solid wastes can reduce the *Aedes* density and dengue cases in Delhi city.

Based on the results of the study, a three pronged strategy was formulated and disseminated to Delhi Municipal Corporation and State governments for control Dengue.

1. Firstly, regular and sustained vector surveillance throughout the year i.e. Nodal persons should be identified in Government and Private Organizations, RWAs, Educational institutions etc. and should be trained for identification and removal of *Aedes* breeding habitats.
2. Secondly, domestic containers play a crucial role in *Aedes* breeding especially during non-transmission season. It is the responsibility of community to clean OHTs, coolers, containers, Mud Pots, Flower Pots and Bird pots etc on regular basis. In peri-domestic containers, curing tanks are also peri-domestic containers mainly created by builders/contractors during construction of building and left unattended without demolishing. Builders/ contractors should be responsible for demolishing or mosquito proofing such tanks.
3. Thirdly, sustained community mobilization with special reference to school and RWAs.
4. Recommendations of the study have been accepted by the Municipal Corporation, Delhi for implementation.

## Supporting Information

S1 Fig Consent from Public Health Department of South Delhi Municipal Corporation to conduct action research study. (JPG)

S1 Table Month and Season-wise larval indices (DOCX)

S2 Table Month, season and container-wise container index (DOCX)
