# Supplementary figures and images for: Control of Aedes aegypti Breeding: A Novel Intervention for Prevention and Control of Dengue in an Endemic Zone of Delhi, India
Source: PLoS One. 2016 Dec 5;11(12):e0166768. doi: 10.1371/journal.pone.0166768 (PMC5137876; doi:10.1371/journal.pone.0166768)

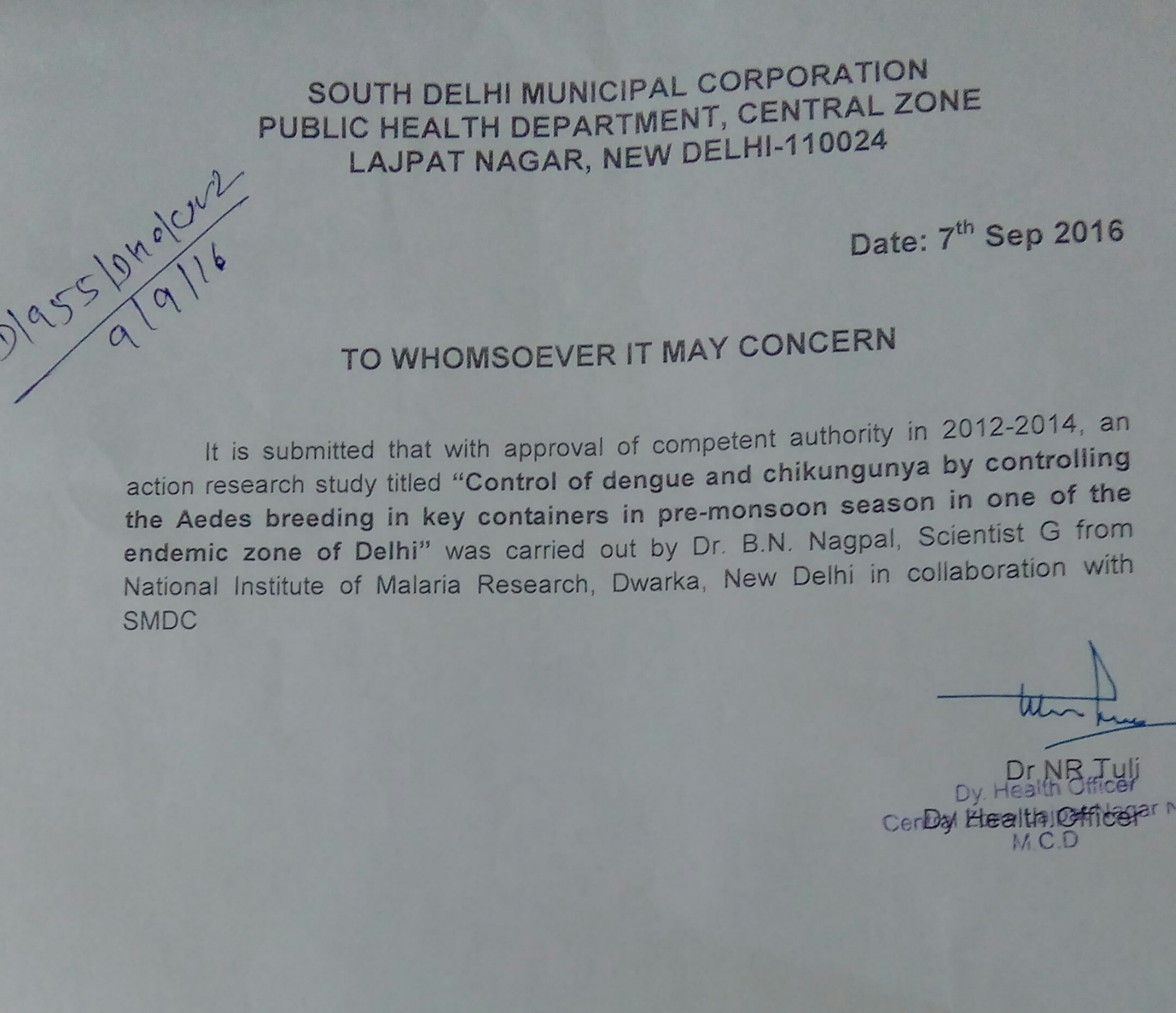

Supplement: S1 Fig — (JPG) [file pone.0166768.s001.jpg]
