# Supplementary material for: Control of Aedes aegypti Breeding: A Novel Intervention for Prevention and Control of Dengue in an Endemic Zone of Delhi, India
Source: PLoS One. 2016 Dec 5;11(12):e0166768. doi: 10.1371/journal.pone.0166768 (PMC5137876; doi:10.1371/journal.pone.0166768)
Supplement: S1 Table — (DOCX) [file pone.0166768.s002.docx]

**Table 1. Month and season-wise larval indices**

| **Season** | **Month** | **HI** | **CI** | **BI** | **PI** |
| --- | --- | --- | --- | --- | --- |
| Transmission Season | Jul,12 | 6.96 | 3.63 | 8.04 | 11.54 |
|  | Aug,12 | 5.48 | 3.10 | 7.59 | 10.12 |
|  | Sep,12 | 3.98 | 2.04 | 4.86 | 5.01 |
|  | Oct,12 | 1.88 | 1.17 | 2.39 | 0.93 |
|  | Nov,12 | 1.01 | 0.73 | 1.43 | 0.52 |
| Non-Transmission Season | Dec,12 | 0.59 | 0.44 | 0.76 | 0.20 |
|  | Jan,13 | 0.61 | 0.42 | 0.73 | 0.22 |
|  | Feb,13 | 0.98 | 0.58 | 1.05 | 0.35 |
|  | Mar,13 | 1.12 | 0.70 | 1.30 | 0.38 |
|  | Apr,13 | 1.31 | 0.89 | 1.68 | 0.15 |
|  | May,13 | 0.87 | 0.62 | 1.18 | 0.04 |
| Transmission Season | Jun,13 | 0.80 | 0.56 | 1.09 | 0.04 |
|  | Jul,13 | 2.23 | 1.48 | 2.84 | 0.03 |
|  | Aug,13 | 2.37 | 1.24 | 2.90 | 0.12 |
|  | Sep,13 | 1.21 | 0.61 | 1.40 | 0.01 |
|  | Oct,13 | 0.96 | 0.51 | 1.15 | 0.03 |
|  | Nov,13 | 0.31 | 0.16 | 0.33 | 0.01 |
| Non-Transmission Season | Dec,13 | 0.16 | 0.09 | 0.17 | 0.01 |
|  | Jan,14 | 0.04 | 0.02 | 0.04 | 0.00 |
|  | Feb,14 | 0.00 | 0.00 | 0.00 | 0.00 |
|  | Mar,14 | 0.03 | 0.02 | 0.03 | 0.00 |
|  | Apr,14 | 0.09 | 0.05 | 0.09 | 0.00 |
|  | May,14 | 0.00 | 0.00 | 0.00 | 0.00 |
