# Supplementary material for: Control of Aedes aegypti Breeding: A Novel Intervention for Prevention and Control of Dengue in an Endemic Zone of Delhi, India
Source: PLoS One. 2016 Dec 5;11(12):e0166768. doi: 10.1371/journal.pone.0166768 (PMC5137876; doi:10.1371/journal.pone.0166768)
Supplement: S2 Table — (DOCX) [file pone.0166768.s003.docx]

**Table 2. Month, season and container-wise container index**

| **Season** | **Month** | **Over Head Tanks** | **Coolers** | **Curing Tanks** | **Mud Pot** | **Solid Waste** | **Water Storage Containers** |
| --- | --- | --- | --- | --- | --- | --- | --- |
| Transmission Season | Jul,12 | 0.51 | 7.14 | 48.57 | 8.49 | 0.00 | 2.50 |
|  | Aug,12 | 0.90 | 5.03 | 32.35 | 5.83 | 14.75 | 3.12 |
|  | Sep,12 | 0.56 | 3.86 | 30.77 | 4.00 | 9.30 | 2.10 |
|  | Oct,12 | 0.39 | 1.37 | 4.67 | 2.67 | 2.94 | 1.52 |
|  | Nov,12 | 0.28 | 11.11 | 5.98 | 1.56 | 0.00 | 0.84 |
| Non-Transmission Season | Dec,12 | 0.32 | 0.00 | 17.14 | 0.90 | 0.00 | 0.35 |
|  | Jan,13 | 0.29 | 25.00 | 22.58 | 0.82 | 20.00 | 0.33 |
|  | Feb,13 | 0.44 | 12.90 | 15.91 | 1.07 | 2.67 | 0.35 |
|  | Mar,13 | 0.41 | 0.00 | 12.90 | 1.52 | 0.00 | 0.75 |
|  | Apr,13 | 0.52 | 1.28 | 6.38 | 1.73 | 0.00 | 1.13 |
|  | May,13 | 0.07 | 3.38 | 5.56 | 0.89 | 0.00 | 0.46 |
| Transmission Season | Jun,13 | 0.08 | 2.28 | 0.00 | 0.75 | 0.00 | 0.46 |
|  | Jul,13 | 0.22 | 2.98 | 28.30 | 3.04 | 14.81 | 1.67 |
|  | Aug,13 | 0.26 | 2.32 | 26.32 | 1.76 | 10.53 | 1.54 |
|  | Sep,13 | 0.15 | 2.23 | 7.04 | 0.76 | 0.00 | 0.59 |
|  | Oct,13 | 0.19 | 2.75 | 15.79 | 0.67 | 100.00 | 0.70 |
|  | Nov,13 | 0.18 | 0.00 | 12.50 | 0.13 | 0.00 | 0.08 |
| Non-Transmission Season | Dec,13 | 0.08 | 0.00 | 27.27 | 0.00 | 0.00 | 0.07 |
|  | Jan,14 | 0.04 | 0.00 | 0.00 | 0.00 | 0.00 | 0.00 |
|  | Feb,14 | 0.00 | 0.00 | 0.00 | 0.00 | 0.00 | 0.00 |
|  | Mar,14 | 0.00 | 50.00 | 0.00 | 0.00 | 0.00 | 0.02 |
|  | Apr,14 | 0.01 | 0.93 | 33.33 | 0.00 | 0.00 | 0.00 |
|  | May,14 | 0.00 | 0.00 | 0.00 | 0.00 | 0.00 | 0.00 |
